# Functional analysis of genes enriched in male reproductive organs: validation via fertility assessment of 14 knockout mouse lines

**DOI:** 10.3389/fcell.2026.1851315

**Published:** 2026-05-28

**Authors:** Kazumasa Takemoto, Katarzyna Kent, Kaori Nozawa, Shingo Tonai, Ahn H. Pham, Terumi Fujimoto, Thomas X. Garcia, Martin M. Matzuk, Masahito Ikawa, Keizo Tokuhiro

**Affiliations:** 1 Department of Genome Editing, Institute of Biomedical Science, Kansai Medical University, Hirakata, Japan; 2 Department of Pathology and Immunology, Baylor College of Medicine, Center for Drug Discovery, Houston, TX, United States; 3 Department of Pathology and Immunology, Baylor College of Medicine, Houston, TX, United States; 4 Department of Molecular and Human Genetics, Baylor College of Medicine, Houston, TX, United States; 5 Department of Experimental Genome Research, Research Institute for Microbial Diseases, The University of Osaka, Suita, Japan; 6 Graduate School of Pharmaceutical Sciences, The University of Osaka, Suita, Japan; 7 Division of Microbiology and Immunology, Center for Infectious Disease Education and Research, The University of Osaka, Suita, Japan; 8 Laboratory of Reproductive Systems Biology, The Institute of Medical Science, The University of Tokyo, Tokyo, Japan

**Keywords:** CRISPR/Cas9, epididymis, evolutionary conservation, gene redundancy, male infertility, null mutant mice, spermatozoa, testis

## Abstract

Tissue-restricted gene expression serves as a key indicator of functional importance within specific organs. Despite advances in high-throughput sequencing and genome editing over the past decade, this principle remains the gold standard for identifying candidate genes for functional studies. In reproductive biology, testis-restricted expression is anticipated to indicate genes crucial for gametogenesis, given the conserved features shared between spermatogenesis and oogenesis. Concurrently, rapid evolution of spermatogenesis-related genes is well documented and thought to arise through duplication and subsequent mutation of ubiquitously expressed genes. In this study, we established 14 knockout (KO) mouse lines for male reproductive organ-enriched genes, all of which exhibited normal growth and fertility in natural mating assays. These genes share sequence similarity with previously characterized gene families or functionally defined paralogs. These observations indicate that while these genes acquired enriched expressions in male reproductive organ following their evolutionary origin, they may represent: (1) currently non-functional loci, (2) genes with redundant functions compensated by paralogs, or (3) genes undergoing loss of function.

## 1 Introduction

Gametogenesis is fundamental to the perpetuation of species. In animals, spermatogenesis and oogenesis necessitate stringent spatiotemporal regulation of gene expression, given the highly organized, sequential stages of cellular differentiation involved (Cardoso-Moreira et al., 2019). Specifically, spermatogenesis in testes encompasses the entire spectrum of cellular differentiation from spermatogonial stem cells to spermatozoa, whereas oogenesis involves dramatic oocyte growth surrounded by layers of somatic granulosa cells, with the zona pellucida forming between the oocyte and granulosa cells (Larose et al., 2019). Nevertheless, spermatogenesis and oogenesis share key features, including the primordial germ cell state and meiosis. Consequently, testis-enriched genes such as *Nanos3*, *Dnd1*, *Stra8* and *Meiosin* frequently prove essential for both gametogenic processes (Tsuda et al., 2003; Ruthig et al., 2019; Youngren et al., 2005; Mark et al., 2008; Baltus et al., 2006; Ishiguro et al., 2020).

The genes implicated in spermatogenesis include both ubiquitously expressed genes, as germ cells require basic somatic cellular functions for survival, and spermatogenesis-specific genes, which are frequently identified as testis-enriched (White-Cooper and Bausek, 2010). Testis-specific genes are hypothesized to arise through two primary mechanisms: (1) duplication of ancestral genes through chromosomal duplication or retroposition, or (2) *de novo* gene birth (White-Cooper and Bausek, 2010; Dorus et al., 2008; Gubala et al., 2017). Following their emergence through duplication from ancestral genes or *de novo* birth, these genes are thought to acquire testis-specific functions (White-Cooper and Bausek, 2010).

Consistent with these concepts, the rapid evolution of the testicular transcriptome has been documented across various studies (Soumillon et al., 2013; Necsulea et al., 2014). To date, 2,375 genes have been identified as spermatogenic cell-restricted ones in the mouse genome; however, KO mouse studies have revealed fertility phenotypes for only 242 genes, suggesting that not all testis-specific genes are functionally indispensable (Schultz et al., 2003; Robertson et al., 2020; Yamamoto et al., 2024). Given the ongoing emergence of testis-specific genes, the rapid evolution of the testicular transcriptome likely generates new genes that are either not yet functionally essential or remain functionally redundant.

While genes lacking phenotypes in deficient mice may seem less informative, comprehensive documentation of such genes is critical for efficient experimental design and cost-effective research strategies (Yamamoto et al., 2024; Miyata et al., 2016; Chang et al., 2026; Nguyen et al., 2024). Moreover, like Rosa26, these genes are sometimes repurposed as experimental tools, such as Cre drivers or GFP reporters—many of which were originally identified through promoter trap screens in embryonic stem cells designed to identify and mutate developmental genes (Friedrich and Soriano, 1991). Here, we report a collection of male reproductive organ-enriched genes that are paralogous to functionally characterized genes but exhibit no fertility defects when disrupted. The existence of these genes suggests several possibilities: evolutionary novelty, functional redundancy, loss of function following emergence, or context-dependent roles whose functional effects under various conditions—such as stress or aging—have yet to be examined.

## 2 Materials and methods

### 2.1 Animals

The animal experiments conducted in this study received approval from the Institutional Animal Care and Use Committees of Baylor College of Medicine (Houston, Texas, USA), University of Osaka (Osaka, Japan), and Kansai Medical University (Osaka, Japan), in strict accordance with the established guidelines and regulations for animal experimentation. The B6D2F1 and ICR mice utilized in this research were procured from CLEA Japan, Inc. (Tokyo, Japan), Japan SLC Inc. (Shizuoka, Japan), or Shimizu Laboratory Supplies (Kyoto, Japan). All gene-modified mice in this study were generated on a hybrid genetic background of B6D2F1, and these mutant mice will be made available to other researchers through either the RIKEN BioResource Research Center, Ibaraki, Japan, or the Center for Animal Resources and Development (CARD), Kumamoto University, Kumamoto, Japan (Supplementary Table S1).

### 2.2 *In silico* PCR

A semi-quantitative *in silico* PCR analysis, presenting values of transcripts per million (TPM) per tissue per gene in both human and mouse reproductive and non-reproductive tissues, was performed using the Mammalian Reproductive Genetics Database V2 (MRGDV2) website (https://orit.research.bcm.edu/MRGDv2) (Robertson et al., 2020). We set the minimum and maximum TPM values to 0 and 30, respectively, to depict *in silico* PCR images in various tissues using MRGDV2.

### 2.3 Reverse transcription PCR (RT-PCR)

Total RNA was extracted from various tissues of wild-type mice utilizing ISOGEN II (NIPPON GENE, Tokyo, Japan) in accordance with the standard protocol. Subsequently, the total RNA was reverse-transcribed into complementary DNA (cDNA) using the PrimeScript RT Reagent Kit (Takara Bio, Shiga, Japan). The polymerase chain reaction (PCR) primers employed for each gene are detailed in Supplementary Table S2.

### 2.4 Generation of KO mice with the CRISPR/Cas9 system

All gene-deficient mouse lines utilized in this study were generated employing the CRISPR/Cas9 system. The CRISPRdirect software was employed to identify off-target sequences during the selection of guide RNAs (Naito et al., 2015) (Supplementary Table S3). To prevent truncated protein expression and eliminate protein function, we intentionally targeted nearly complete coding regions of each gene locus. The generation of gene-deficient mice was achieved through electroporation using zygotes, as previously described (Noda et al., 2019; Abbasi et al., 2018). The crRNA and tracrRNA (Integrated DNA Technologies, Coralville, IA, USA or Merck, Darmstadt, Germany) were mixed and hybridized by heating to 95 °C and subsequently cooling to room temperature in duplex buffer (Integrated DNA Technologies). The Cas9 protein (Thermo Fisher Scientific, Waltham, MA, USA or Integrated DNA Technologies) was combined with the hybridized RNAs in Opti-MEM (Thermo Fisher Scientific). This solution was incubated at 37 °C to form the gRNA/Cas9 ribonucleoprotein (RNP) complex, which was electroporated into fertilized eggs using a NEPA21 Super Electroporator (Nepagene, Chiba, Japan). Embryos that progressed to the 2-cell stage were transplanted into the oviducts of pseudopregnant ICR females at 0.5 days post-mating with vasectomized males. Pups (Founder 0) were delivered naturally or by cesarean section, and F0 mice harboring each mutation were mated with wild-type B6D2F1 mice from a commercial source to produce heterozygous F1 offspring. Subsequently, F1 heterozygotes were interbred to produce F2 homozygous mutated offspring. All experiments were conducted using F2 or F3-generation mice for analysis. Genotyping was performed using PCR with the primers listed in Supplementary Table S4. To examine the deleted region in the mutated allele of each line, we purified the amplified PCR products using the respective primers that detected mutated alleles. Subsequently, the samples were subjected to Sanger sequencing.

### 2.5 Morphological and histological analysis of testes

Following the measurement of whole body and testicular weights, the testes were fixed in Bouin’s solution (Polysciences, Warrington, PA, USA or FUJIFILM Wako, Osaka, Japan), embedded in paraffin, and sectioned to a thickness of four or 5 μm using either a Microm HM325 microtome (Microm, Walldorf, Germany) or an RM2125RT microtome (Leica, Nussloch, Germany). The sections were then rehydrated and treated with 1% periodic acid for 10 min. Subsequently, Schiff’s reagent (FUJIFILM Wako) was applied for 20 min. The periodic acid–Schiff stained sections were further stained with Mayer’s hematoxylin solution (FUJIFILM Wako). Observations were conducted using an Olympus BX53 microscope equipped with an Olympus DP74 color camera (Olympus, Tokyo, Japan) or a BZ-X810 (Keyence, Osaka, Japan).

### 2.6 Analysis of morphology and motility of spermatozoa

Spermatozoa obtained from the cauda epididymis were suspended in either TYH or HTF medium (Toyoda et al., 1971; Quinn et al., 1985) and incubated at 37 °C for 30 min. Subsequently, the samples were diluted and placed on MAS-coated glass slides (Matsunami Glass, Osaka, Japan) for morphological assessment using an Olympus BX53 microscope. Sperm numbers and motility parameters in 16-week-old WT and KO male mice (n = 5) were examined using CASA. Cauda epididymides were minced in HTF medium and incubated at 37 °C. Sperm supernatant was diluted 1:50 in HTF, added to a pre-warmed counting slide, and analyzed with the CEROS II system (Hamilton Thorne Biosciences, Beverly, MA, USA). At least 200 cells were counted.

### 2.7 Fertility analysis of KO lines

Sexually mature control or KO male mice were housed individually with one to three 8-week-old wild-type B6D2F1 female mice in the same cage for a minimum duration of 8 weeks. To ensure statistical robustness, three to five males were evaluated for each KO line. Upon conclusion of the mating period, the male mice were removed from the cages, while the wild-type females were retained for an additional 3 weeks to facilitate the delivery of their final litters.

### 2.8 *In vitro* fertilization

Spermatozoa harvested from the cauda epididymis were incubated in a drop of mHTF medium for 1 hour at 37 °C under 5% CO2 conditions (Kito et al., 2004). For egg collection, B6D2F1 females received intraperitoneal injections of 7.5 IU pregnant mare serum gonadotropin (PMSG; ASKA Pharmaceutical, Tokyo, Japan) followed 48 h later by 7.5 IU human chorionic gonadotropin (hCG; ASKA Pharmaceutical, Tokyo, Japan). Eggs were subsequently retrieved from the superovulated females 14 h post-hCG injection. Spermatozoa were introduced to the mHTF drops containing cumulus-intact eggs at a final concentration of 2 × 10^5^ spermatozoa/mL and incubated at 37 °C under 5% CO2. Following a five-hour insemination period, the eggs were transferred to a drop of KSOM medium (Lawitts and Biggers, 1993). The subsequent day, two-cell embryos were counted.

### 2.9 Statistical analyses

Statistical significance was assessed using a two-tailed unpaired Student’s t-test, with the assumption of unequal variances, conducted via Microsoft Office Excel (Microsoft Corporation, Redmond, WA, USA). A P-value of less than 0.05 was considered indicative of statistical significance. Data are presented as means ± standard deviation (SD) or standard error of the mean (SEM).

## 3 Result

### 3.1 *In silico* expression analyses of reproductive organ-enriched genes

**FIGURE 1:**
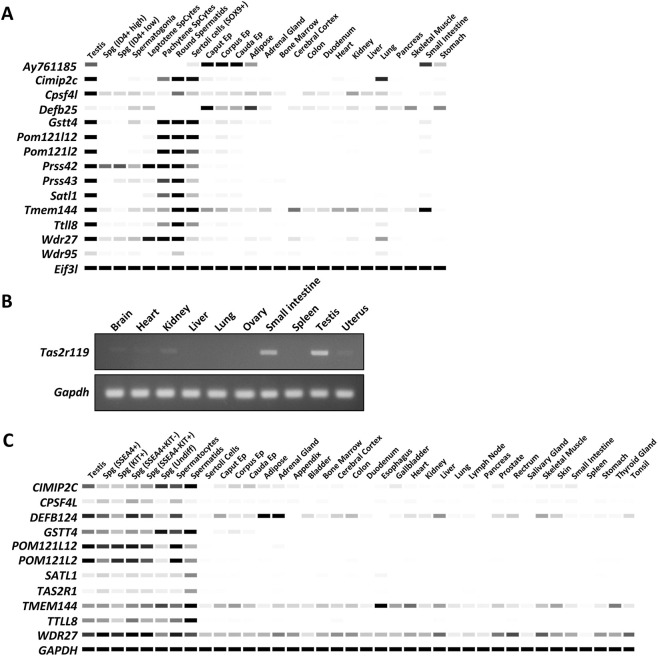
*In silico* analyses of the expression patterns of specific genes in multiple tissues and spermatogenic cells. **(A)** *In silico* PCR indicates the expression patterns of 14 genes of interest in mouse tissues utilizing the Mammalian Reproductive Genetics Database V2 (MRGDV2) website. *Cpsf4l* and *Tmem144* are detected slightly weak signals in ubiquitous tissues and highly expressed in testis. The other 12 genes showed predominant or restricted expression in mouse testes or epididymides. White = 0 TPM, Black ≥30 TPM. *Eif3l* is used as an internal control. *Prss44*-deficient mice were previously reported by our group and the expression data were not included in this figure (Holcomb et al., 2020). **(B)** RT-PCR indicates the expression patterns of *Tas2r119* in mouse tissues. *Gapdh* is used as an internal control. **(C)** Digital PCR indicates the expression patterns of 11 genes of interest in human tissues. White = 0 TPM, Black ≥30 TPM. *GAPDH* is used as an internal control. *DEFA1*, *DEFA1B*, and *DEFA3* are orthologs of *Ay761185*, with no expression data available from *in silico* analysis. *Prss42*, *Prss43*, and *Wdr95* are not found in human genomes.

Utilizing an *in silico* PCR system, we identified genes that are either exclusively or highly specific to the male reproductive organ (Figure 1). These genes exhibited robust expression in the testis or epididymis, as well as in a limited number of other organs (Figure 1A). Given the lack of *in silico* expression data for *Tas2r119*, we conducted RT-PCR analysis across mouse tissues, which revealed high expression in the small intestine and testis (Figure 1B). Several genes (*Prss42*, *Prss43*, and *Wdr95*) lack human orthologs, whereas the remaining genes show predominantly reproductive organ-enriched expression with limited expression in select additional tissues (Figure 1C). Moreover, orthologous genes in the mouse or human genome were identified for all these genes, some of which are significant for non-spermatogenesis-related factors. For instance, *Sat1* (spermidine/spermine N1-acetyltransferase 1), a paralog of *Satl1*, is associated with late-onset obesity in fat-specific conditional KO mice (Yuan et al., 2018). CPSF4L is a paralog of CPSF4 (cleavage and polyadenylation specific factor 4), which plays a crucial role in pre-mRNA modification (Clerici et al., 2017). The expression patterns and presence of these paralogous genes suggest functional divergence toward male reproductive organ-specific roles.

### 3.2 Production and phenotypic analyses of gene-deficient mice

**FIGURE 2:**
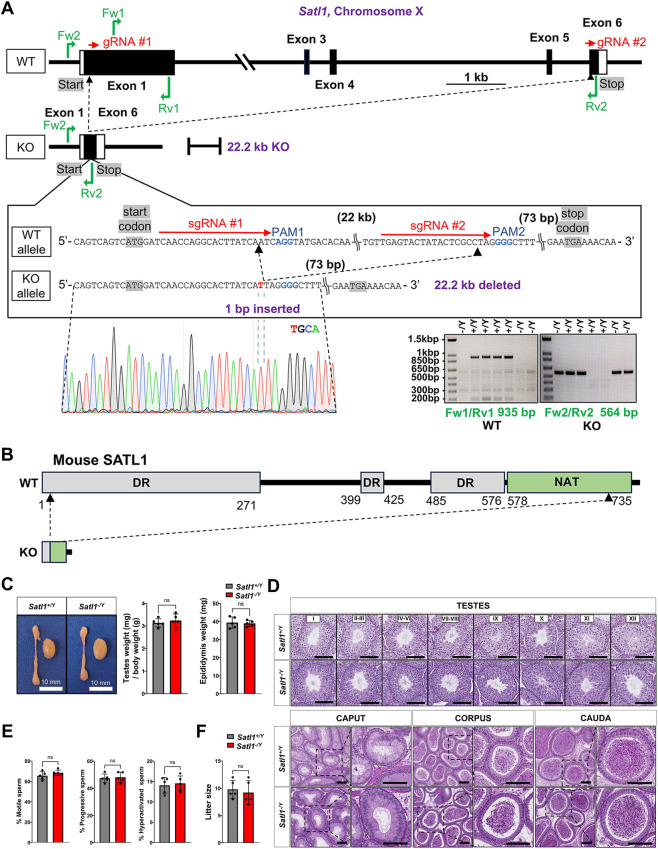
Phenotypic analysis of *Satl1* deficient male mice. **(A)** A schematic representation of the genomic locus of *Satl1* on mouse chromosome X is provided. Exons involved in gene deletion are marked, with the corresponding locations of each guide RNA (gRNA), Cas9 cleavage sites, and PAM sequences indicated by red arrows. Genotyping primers, forward (Fw) and reverse (Rv), and their orientations are denoted by green arrows. The size of the gene deletion is specified in kilobases (kb). Sanger sequencing confirms a 22.2 kb deletion with a 1 bp insertion (highlighted in red). PCR results verify the hemizygous deletion of Satl1 in genomic DNA from the F2 generation, represented by the 564 bp band amplified by primer set 2 (Fw2/Rv2). A 1 kb scale is provided for reference. **(B)** A diagram of the SATL1 protein structure in wild-type (WT) and hemizygous KO mice is shown. Black arrows indicate the position of Cas9 cleavage sites in the coding sequence of SATL1, resulting in a truncated protein in the hemizygous KO mice. The relative positions of regions and domains are indicated by the number of amino acids along the protein sequence. DR = disordered region, NAT = N-acetyltransferase domain. **(C)** Testis and epididymis weight normalized to body weight, indicating no structural abnormalities in *Satl1* KO males. **(D)** Histological analysis of PAS-stained sections of testes and epididymides from hemizygous *Satl1* WT and KO mice. Scale bar = 100 µm. **(E)** Sperm count in cauda epididymides indicates comparable sperm concentration and sperm motility parameters between groups. The percentage of motile, progressive, and hyperactive sperm demonstrates normal motility parameters in *Satl1* deficient mice. **(F)** Litter size and count per male per month from breeding trials with two WT females per male. Data are presented as mean ± SEM. Statistical significance was assessed using Student’s t-test (n = 5 per group). ns indicates no significant difference.

**FIGURE 3:**
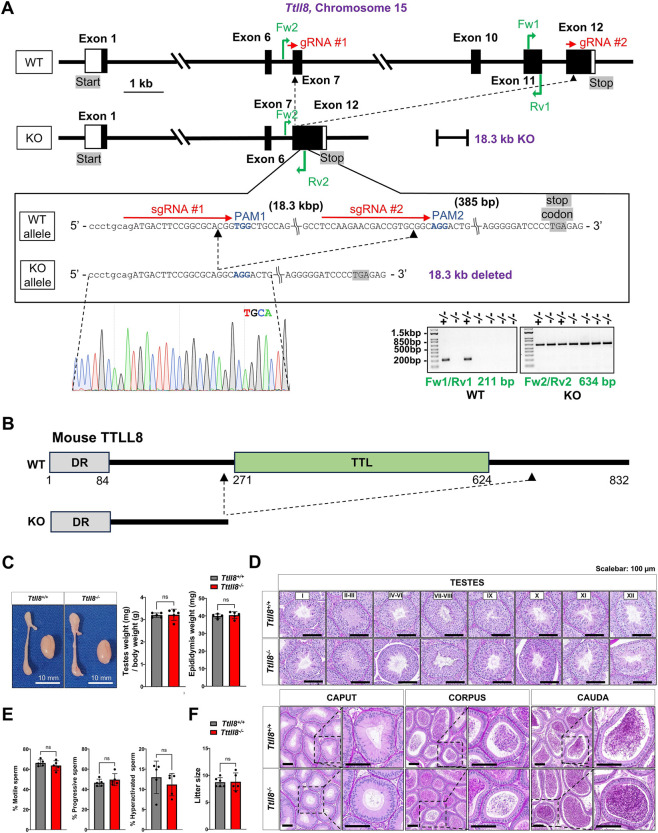
Phenotypic analysis of *Ttll8*-deficient male mice. **(A)** A schematic representation of the genomic locus of *Ttll8* on mouse chromosome 15 is provided. Exons involved in the gene deletion are marked, along with the locations of each guide RNA (gRNA), Cas9 cleavage sites, and PAM sequences, indicated by red arrows. Genotyping primers, forward (Fw) and reverse (Rv), and their orientations are denoted by green arrows. The size of the gene deletion is specified in kilobases (kb). Sanger sequencing confirms an 18.3 kb deletion. PCR results verify the hemizygous deletion of *Ttll8* in genomic DNA from the F2 generation, represented by the 634 bp band amplified by primer set 2 (Fw2/Rv2). A 1 kb scale is provided for reference. **(B)** A diagram of the TTLL8 protein structure in wild-type (WT) and homozygous KO mice is shown. Black arrows indicate the position of Cas9 cleavage sites in the coding sequence of TTLL8, resulting in a truncated protein in the homozygous KO mice. The relative positions of regions and domains are indicated by the number of amino acids along the protein sequence. DR = disordered region, TTL = tubulin tyrosine ligase domain. **(C)** Gross morphology of reproductive organs from homozygous *Ttll8* KO (red) and WT mice (gray) is presented. Testis and epididymis weight normalized to body weight, indicating no structural abnormalities in *Ttll8* KO males. **(D)** Histological analysis of PAS-stained sections of testes and epididymides from homozygous *Ttll8* WT and KO mice. Scale bar = 100 µm. **(E)** Sperm count in cauda epididymides indicates comparable sperm concentration and sperm motility parameters between groups. The percentage of motile, progressive, and hyperactive sperm demonstrates normal motility parameters in *Ttll8* KO mice. **(F)** Litter size and count per male per month from breeding trials with two WT females per male. Data are presented as mean ± SEM. Statistical significance was assessed using Student’s t-test (n = 5 per group). ns indicates no significant difference.

**FIGURE 4:**
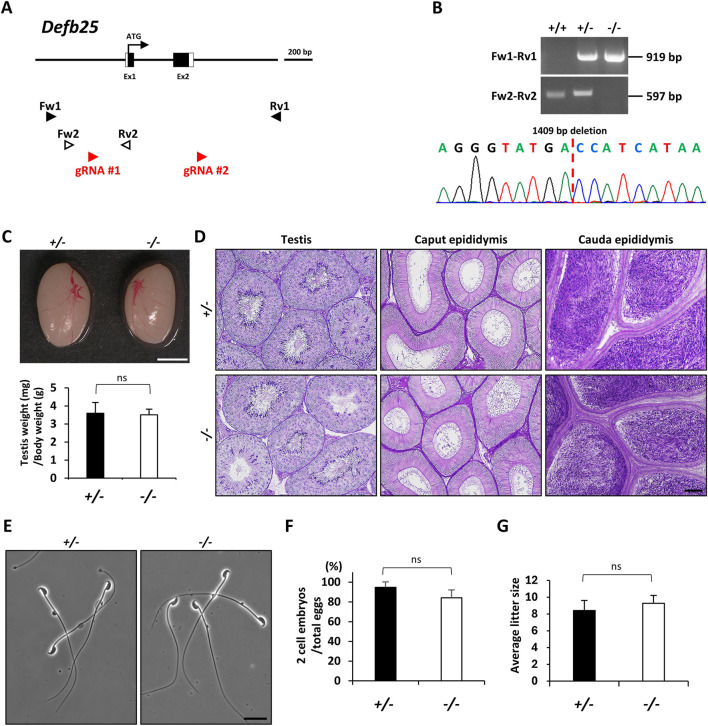
Phenotypic analysis of *Defb25* deficient male mice. **(A)** Genomic structure and KO strategy of *Defb25*. Two guide RNAs were designed to target the 5′ region of first coding exon (Exon 1) and 3′ region of Exon 2. Four primers (Fw1, 2 and Rv1, 2) were designed for genotyping the mutant mice. **(B)** Mutant and wild-type alleles were detected by genomic PCR using primer sets Fw1-Rv1 (mutant allele) and Fw2-Rv2 (WT allele). DNA sequence and chromatograph of the mutant allele by Sanger sequencing were shown in the lower panel. **(C)** Testis appearance and testis to body weight ratios of *Defb25* heterozygous and homozygous mutated mice. Scale bar = 3 mm. **(D)** Histological analysis of testes and epididymides in control and *Defb25* deficient mice. Scale bar = 50 μm. **(E)** Morphology of cauda epididymal spermatozoa in control and *Defb25* deficient mice. Scale bar = 20 μm. **(F)** *In vitro* fertilization with cumulus-intact oocytes. **(G)** Average litter size of control and *Defb25* deficient male mice. ns indicates not significant.

**FIGURE 5:**
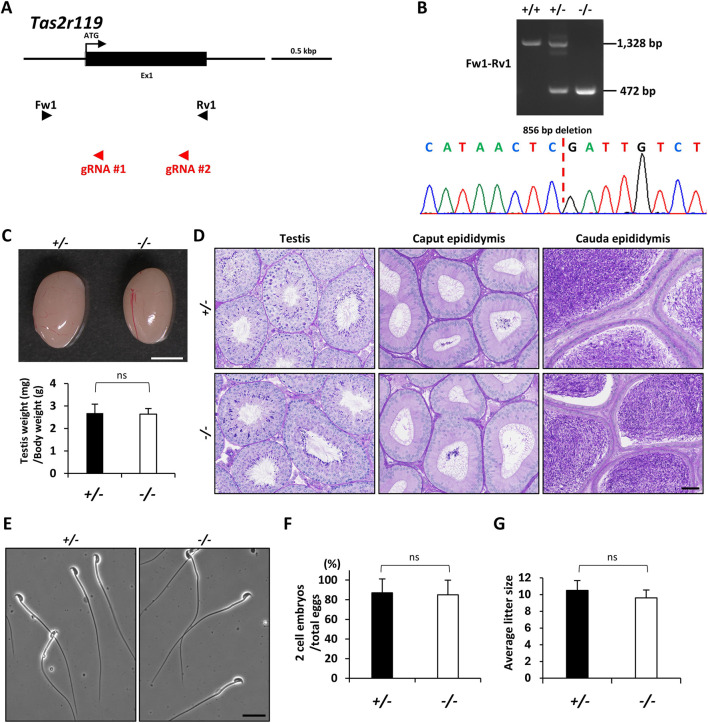
Phenotypic analysis of *Tas2r119* deficient male mice. **(A)** Genomic structure and KO strategy of *Tas2r119*. Two guide RNAs were designed to target the first coding exon (Exon 1). Two primers (Fw1 and Rv1) were designed for genotyping the mutant mice. **(B)** Mutant and wild-type alleles were detected by genomic PCR using the primer set Fw1-Rv1 (mutant and WT allele). DNA sequence and chromatograph of the mutant allele by Sanger sequencing were shown in the lower panel. **(C)** Testis appearance and testis to body weight ratios of *Tas2r119* heterozygous and homozygous mutated mice. Scale bar = 3 mm. **(D)** Histological analysis of testes and epididymides in control and *Tas2r119* deficient mice. Scale bar = 50 μm. **(E)** Morphology of cauda epididymal spermatozoa in control and *Tas2r119* deficient mice. Scale bar = 20 μm. **(F)** *In vitro* fertilization with cumulus-intact oocytes. **(G)** Average litter size of control and *Tas2r119* deficient male mice. ns indicates not significant.

We utilized the CRISPR/Cas9 system to generate KO alleles by inducing large deletions with dual guide RNAs, thereby eliminating most coding sequences or complete duplicated gene clusters (Figures 2A–5A; Supplementary Figure S1). For genes with typical exon-intron structures, such as *Cimip2c*, *Cpsf4l*, *Gstt4*, *Satl1*, *Ttll8* and *Tmem144* the majority of exons were depleted (Figures 2A, 3A; Supplementary Figure S1). However, for *Wdr27* and *Wdr95*, we avoided targeting complete coding regions to prevent deletion of piRNA clusters located in introns. In the cases of genes with only one or two exons, including *Ay761185*, *Defb25*, *Tas2r119*, *Pom121l12*, and *Pom121l2*, the entire gene locus or the first exon was depleted (Figures 4A, 5A; Supplementary Figure S1). *Prss42*, *Prss43* and *Prss44* form a contiguous gene cluster on the same chromosome; therefore, triple KO mice were generated through deletion of the entire cluster (Supplementary Figure S1).

In *Satl1*, deletion of exons two to five in their entirety, together with partial deletions of exons 1 and 6, resulted in elimination of the most disordered domain and the N-acetyltransferase domain (Figures 2A,B). Despite testis-specific expression in both mice and humans, *Satl1* deficient males exhibited no discernible differences from the control in overall morphology, testis-to-body weight ratio, or histological appearance of testicular and epididymal tissues (Figures 2C,D). Furthermore, the proportions of motile, progressive, and hyperactivated sperm in the cauda epididymis were indistinguishable between control and KO males. Accordingly, KO males displayed normal fertility in natural mating assays, with litter sizes equivalent to those of control mice (Figures 2E,F).

The *Ttll8* KO allele, characterized by the depletion of entire exons 8–11 and partial exons 7 and 12, resulted in the loss of the tubulin-tyrosine ligase domain (Figures 3A,B). *Ttll8* is evolutionarily conserved between humans and mice and exhibits testis-specific expression (Figures 1A,C). Comprehensive phenotypic analysis of KO males revealed no abnormalities in testicular or epididymal morphology, testis-to-body weight ratios, histology, sperm motility parameters, or fertility through natural mating (Figure, 3C-F).

Complete deletion of the *Defb25* locus, which comprises two coding exons, was achieved (Figures 4A,B). *Defb25*, the mouse ortholog of human *DEFB124*, encodes defensin beta-25 (beta-124) and shows predominant expression in the murine epididymis and both testis and epididymis in humans, with notable expression in adipose tissue (Figures 1A,C). Despite its restricted expression pattern, *Defb25* deficient mice were born healthy with testis sizes comparable to heterozygous controls (Figure 4C). Histological analysis revealed no phenotypic differences between KO and control testes or epididymides (Figure 4D). Sperm collected from the cauda epididymis exhibited no significant morphological or functional defects (Figures 4E–G).

*Tas2r119* in mice and *TAS2R1* in humans are single-exon genes encoding taste receptors with predominant testicular expression (Figures 1B,C). We disrupted the mouse *Tas2r119* locus through CRISPR/Cas9-induced partial deletion covering a substantial portion of the coding region (Figures 5A,B). In contrast to the testis-specific expression of human *TAS2R1*, mouse *Tas2r119* exhibits high expression in testis, small intestine, and kidney (Figure 1B). Nevertheless, *Tas2r119* deficient mice developed normally with no observable defects, including testis size (Figure 5C). Histological examination of testicular and epididymal (caput and cauda) tissues from KO mice revealed normal architecture, and morphologically normal mature spermatozoa were recovered from the cauda epididymis (Figures 5D,E). Functional analysis demonstrated that KO sperm retained full fertilization capacity in both *in vitro* fertilization and natural mating (Figures 5F,G).

For the remaining genes—*Ay761185*, *Cimip2c*, *Cpsf4l*, *Gstt4*, *Pom121l12*, *Pom121l2*, the *Prss42*/*Prss43*/*Prss44* cluster, *Tmem144*, *Wdr27* and *Wdr95*—we successfully generated healthy KO mice displaying normal testicular and epididymal histology, as well as normal sperm morphology (Supplementary Figures S1-S4).

### 3.3 Fertility results

When housed individually with wild-type females, gene-deficient males sired offspring with average litter sizes comparable to wild-type controls (Table 1). Thus, all KO males examined in this study displayed normal fertility.

**TABLE 1:** Results of the fertility tests for the 14 KO mouse lines. Each wild-type (WT) or mutant males was individually caged with WT female mice.

Gene	Genotype	Average litter size ±SD	No. of males	No. of delivery	No. of pups	P-value
Wild type	—	8.9 ± 2.7	3	18	161	-
*Ay761185*	−1,441/-1,441	10.6 ± 1.8	3	18	191	0.04
*Cimip2c*	−17,870/-17,870	9.6 ± 2.2	3	22	211	0.44
*Cpsf4l*	−7,942/-7,942	10.6 ± 2.2	3	22	233	0.04
*Defb25*	−1,409/-1,409	9.3 ± 2.1	3	18	167	0.68
*Gstt4*	−7,536/-7,536	8.8 ± 2.8	3	23	203	0.89
*Pom121l12*	−671/-671	9.1 ± 3.3	3	20	183	0.91
*Pom121l2*	−3,331/-3,331	9.1 ± 2.8	3	18	164	0.85
*prss42, prss43, prss44 (Triple KO)*	−32,202/-32,202	8.6 ± 2.0	3	19	163	0.64
*Satl1*	−22,186 + 1/Y	9.9 ± 2.0	5	43	426	0.13
*Tas2r119*	−856/-856	9.6 ± 1.9	3	18	173	0.40
*Tmem144*	−19,945/-19,945	8.9 ± 2.5	3	22	196	0.97
*Ttll8*	−18,332/-18,332	8.6 ± 3.0	5	26	224	0.71
*Wdr27*	−13,916/-13,916	9 ± 1.9	3	17	164	0.94
*Wdr95*	−25,302/-25,302	10.2 ± 1.5	3	18	183	0.11

SD indicates standard deviation. P-values were calculated by comparing litter size data to wild-type controls. The reduction in litter size within each deficient line was not statistically significant compared to wild type. Notably, litter sizes in the *Ay761185* and *Cpsf4l* deficient lines were slightly increased.

## 4 Discussion

Gene KO approaches have served as cornerstone methodologies for functional gene characterization in basic biological and clinical research over recent decades (Müller, 1999). The advent of CRISPR/Cas9 genome editing has revolutionized KO mouse generation, offering superior efficiency, speed, and precision relative to conventional ES cell-based strategies (Kaneko et al., 2014). Nevertheless, functional annotation remains incomplete for a substantial number of genes. We previously conducted a large-scale screen of functionally uncharacterized testis-enriched genes (Yamamoto et al., 2024; Miyata et al., 2016; Chang et al., 2026; Nguyen et al., 2024; Abbasi et al., 2018). Notably, many of these genes, including those analyzed here, failed to produce detectable phenotypes when disrupted (Yamamoto et al., 2024; Miyata et al., 2016; Chang et al., 2026; Nguyen et al., 2024). However, such phenotypic neutrality does not necessarily reflect functional redundancy, as compensatory mechanisms involving paralogous genes may mask essential roles in spermatogenesis.

*Satl1* encodes spermidine/spermine N1-acetyltransferase-like 1 and is paralogous to *Sat1* (Yuan et al., 2018). Human *SAT1* exhibits ubiquitous expression, and loss-of-function mutations are associated with X-linked childhood-onset systemic lupus erythematosus (Xu et al., 2022). In mice, adipose-specific *Sat1* conditional KO leads to late-onset obesity, indicating critical metabolic functions potentially shared with *Satl1* in testicular tissue (Yuan et al., 2018). While our KO mice exhibited no overt phenotype, precluding definitive functional assignment, the testis-restricted expression of *Satl1* suggests evolutionary divergence toward a specialized spermatogenic function (Figure 2).

Consistent with paralogous gene compensation, *Ttll8* KO mice displayed no phenotype (Figure 3). The mammalian *Ttll* enzyme family comprises multiple paralogs, and prior characterization of *Ttll3*/*Ttll8* double KOs demonstrated subfertility resulting from aberrant sperm motility (Garnham et al., 2015; Gadadhar et al., 2021). Mirroring the *Sat1*/*Satl1* paradigm, *Ttll3* exhibits ubiquitous expression whereas *Ttll8* is testis restricted. This expression divergence suggests that *Ttll8* may be undergoing subfunctionalization toward testis-specific roles during rodent genome evolution, reminiscent of *Dmc1*, which diverged functionally from its ubiquitously expressed recombination paralog *Rad51* (Rockmill et al., 1995).

*Defb19*/*119*, a fertility-associated beta-defensin, is conserved between mice and humans (Li et al., 2022). Originally characterized as a Sertoli cell product, *Defb19* is also expressed in the murine oviduct where it functions as a sperm chemoattractant (Li et al., 2022; Yamamoto and Matsui, 2002). Human beta-defensin 1 and 126 are also reported as fertility-associated defensins; β-defensin 1 is involved in the capacitation of acrosomes in the female reproductive tract, and β-defensin 126 is associated with sperm motility and anti-immune system factors (Fesahat et al., 2023). In addition, a large deletion of the β-defensin cluster in mice chromosome 8 leads to alterations in intracellular calcium, spontaneous acrosome reaction, and male infertility (Zhou et al., 2013). Likewise, *Defb25*/*124* exhibits prominent expression in the epididymis and testis, with modest expression in germ cells (Figures 1A,C). Despite this reproductive tract-enriched expression, *Defb25* deficient males show normal fertility (Figure 4). Recently, Single-cell RNA and spatial transcriptome sequencing have revealed that epididymal epithelial cells display region-specific gene expression in different epididymal segments, including the beta-defensin family genes (Zhang et al., 2024). Multiple defensins are presumed to be expressed in a stage-dependent manner throughout sperm maturation, with each epididymal compartment contributing to this process. *Defb15*, *Defb18*, *Defb20*, *Defb25*, and *Defb48* are specifically expressed in the caput epididymis. This phenotypic neutrality in *Defb25*-deficient mice likely reflects functional compensation by other beta-defensin family members. Furthermore, our laboratory-based fertility assessments may not adequately challenge the primary antimicrobial functions of defensins. Supporting a conserved antibacterial role, the beta-defensin homolog *HeBD* exhibits antimicrobial activity in seahorse testis, suggesting that *DEFB25* may similarly serve innate immune functions in mammalian reproductive tissues (Huang et al., 2024).

Bitter taste receptors (Tas2rs) are hypothesized to serve dual roles: as behavioral deterrents against toxic substance ingestion and as chemosensory receptors in gastrointestinal, respiratory, and central nervous tissues. Remarkably, the mRNA expression of 35 Tas2r paralogs were detected in testis (Xu et al., 2013). While two postmeiotic receptors, *Tas2r105* and *Tas2r108*, have been deorphanized, the ligands and biological functions of *Tas2r119* remain uncharacterized (Xu et al., 2013; Chandrashekar et al., 2000). The absence of phenotypes in our *Tas2r119* KO mice precluded definitive functional assignment. Nevertheless, compensation by other testis-expressed *Tas2r* family members represents a plausible explanation for phenotypic neutrality.

Here, we generated 14 KO lines targeting genes with male reproductive tract-enriched or -restricted expression, complementing four previously analyzed loci (Figure 1; Supplementary Figures S1–S4). Notably, none of these KOs—including the *Prss42*/*Prss43*/*Prss44* triple mutant—exhibited fertility defects. Previous work demonstrated that antibody-mediated inhibition of the testis-specific serine proteases *Prss42*/*Prss43* in organ culture triggers apoptosis in primary spermatocytes (Yoneda et al., 2013). The contrast between this phenotype and the viability of our genetic KOs suggests that either compensatory pathways buffer against loss *in vivo*, or the pathway is non-essential under laboratory breeding conditions, or physical suppression of *Prss42*/*Prss43* triggers survival defects that never can be triggered in our mutant. Considering these distinct experimental approaches and paralog presence, genes in this study may represent evolutionary intermediates acquiring spermatogenic functions or provide redundant support within broader molecular networks (White-Cooper and Bausek, 2010). Consistent with our results, among the genes we analyzed, *Ttll8*, *Tas2r119*, *Cimip2c*, *Cpsf4l*, *Pom121l2*, *Tmem144* and *Wdr27* are also reported to have no impact on fertility in the International Mouse Phenotyping Consortium (IMPC, www.mousephenotype.org) (Wilson et al., 2026). Additionally, like *Rosa26*, these loci offer potential safe-harbor sites for expressing reporters (GFP), recombinases (Cre), or genome editing tools (CRISPR/Cas9) in spermatogenic cells (Friedrich and Soriano, 1991). Collectively, while these male reproductive-enriched genes appear dispensable in laboratory settings, they likely contribute to the molecular and evolutionary robustness of spermatogenesis.

## Data Availability

The original contributions presented in the study are included in the article/Supplementary material, further inquiries can be directed to the corresponding authors.
