# Supplementary material for: Functional analysis of genes enriched in male reproductive organs: validation via fertility assessment of 14 knockout mouse lines
Source: Front Cell Dev Biol. 2026 May 28;14:1851315. doi: 10.3389/fcell.2026.1851315 (PMC13253961; doi:10.3389/fcell.2026.1851315)
Supplement: Supplementary file 1 [file DataSheet1.zip › Supplementary figures and tables 1, 2.PDF]

# *Supplementary Material*

## 1 Supplementary Figures and Tables

### 1.1 Supplementary Figures

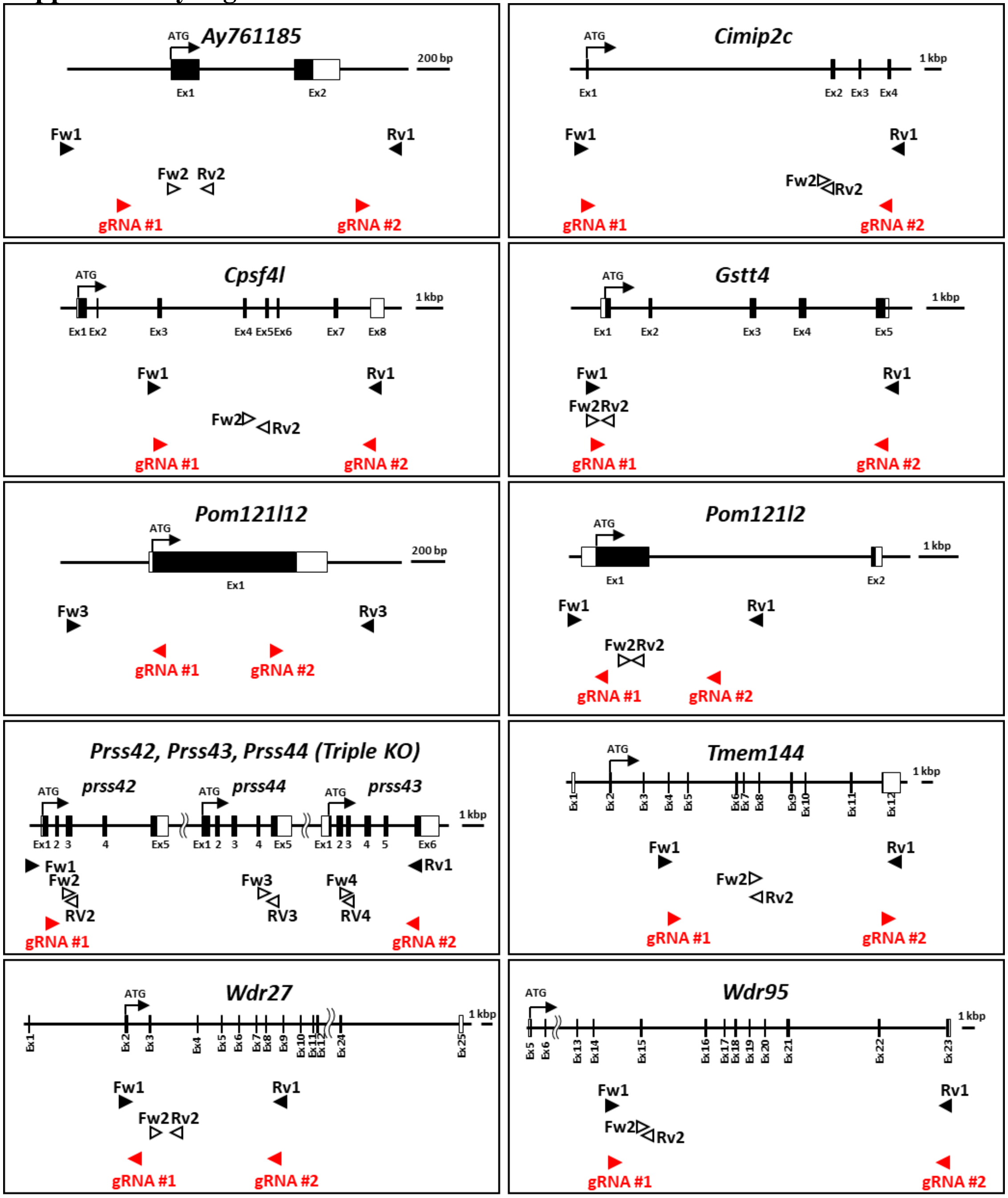


**Supplementary Figure 1.** Schematic representation of the genomic structure and the Knockout strategy in each line.

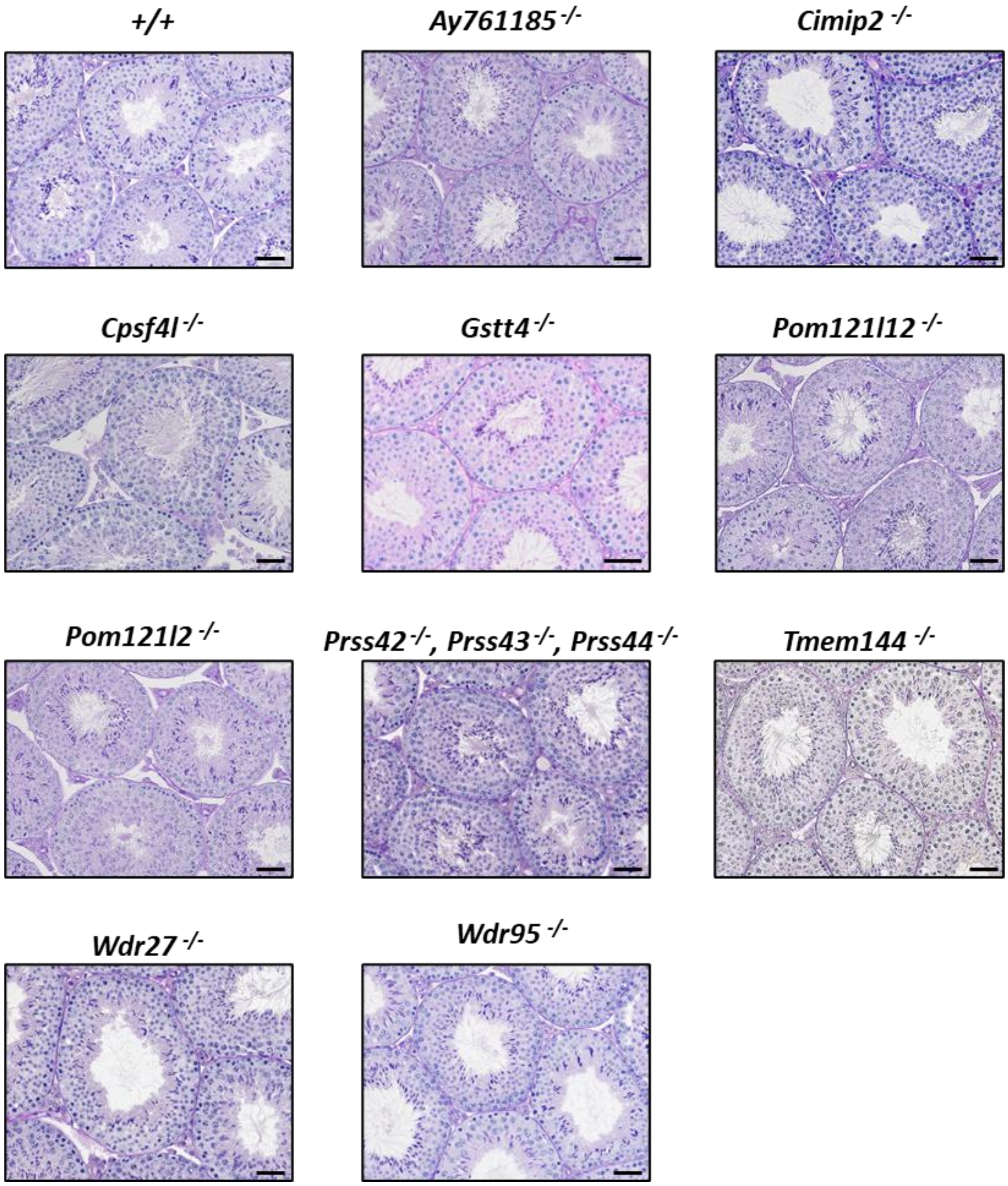


**Supplementary Figure 2.** Histological analysis of testes showed normal morphology in each line. Scale bar = 50 µm.

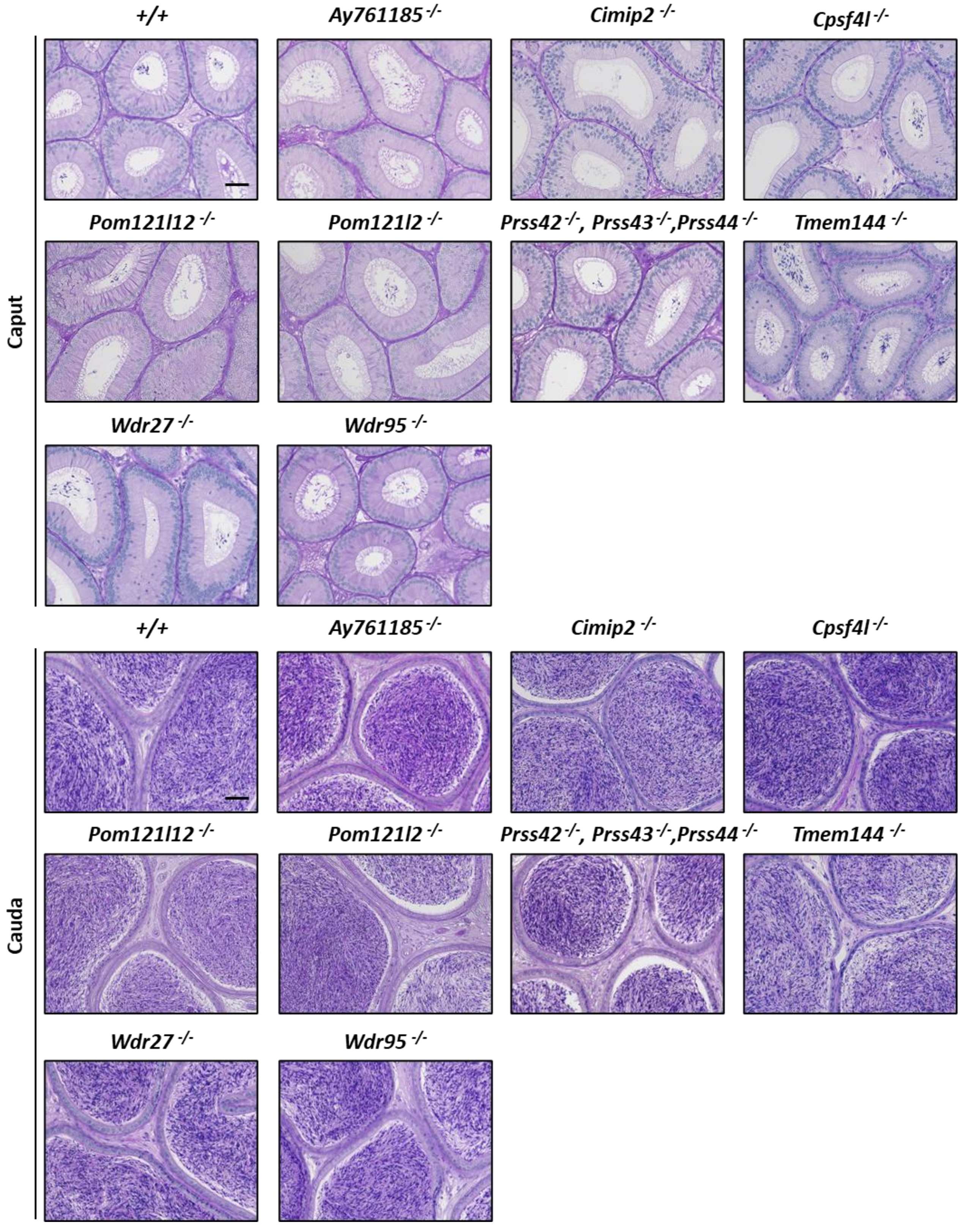


**Supplementary Figure 3.** Epididymal segments (caput and cauda) exhibit normal epithelial structure, with spermatozoa clearly visible in the lumen. Epididymis of *Gstt4* deficient mice were not examined. Scale bar = 50 µm

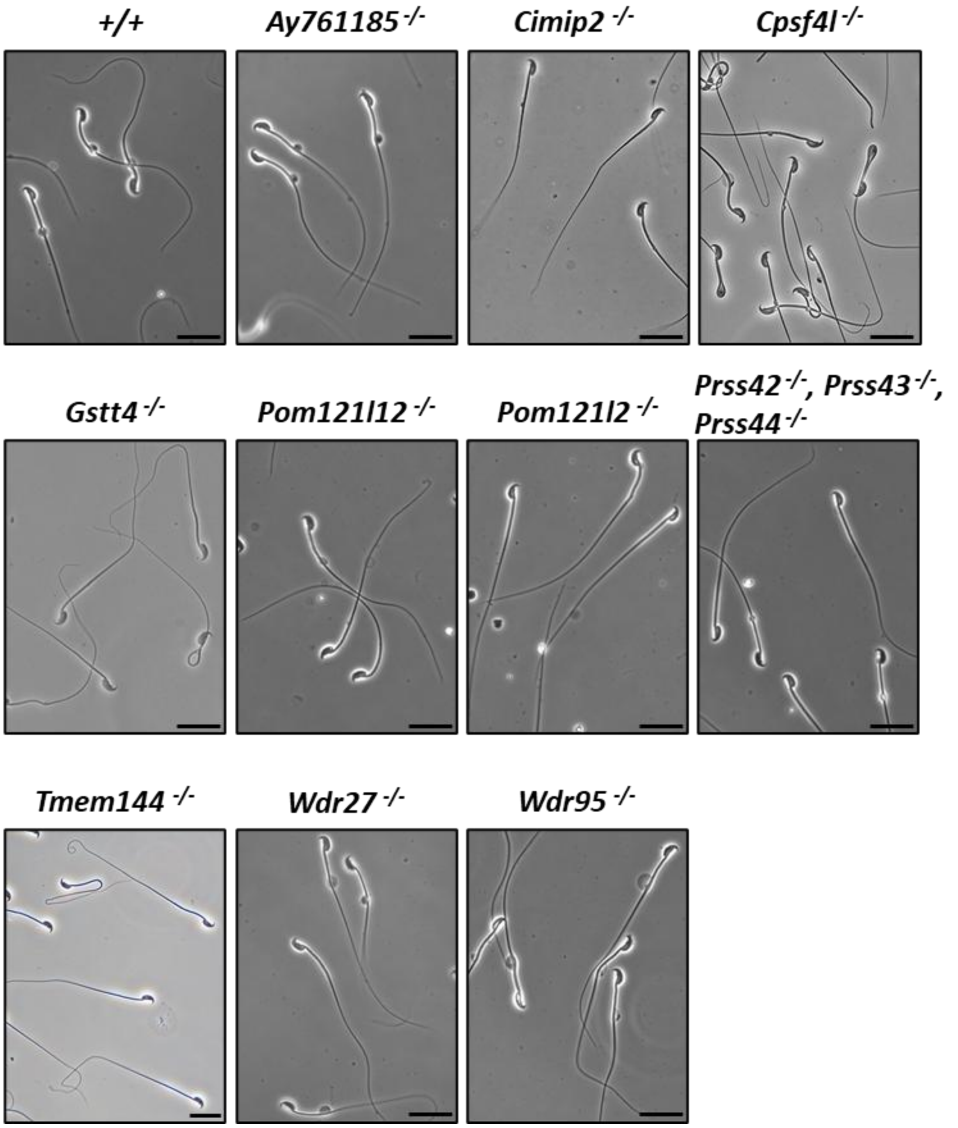


**Supplementary Figure 4.** All sperm isolated from cauda epididymis in each line showed normal morphology. Scale bar = 20 µm.

## 1.2 Supplementary Tables

**Supplementary Table 1.** Bioresource information for the 12 gene-deficient mouse line in this study. The RBRC No. and CARD ID are available for the mouse lines that have been deposited as frozen sperm to Riken Bio reserch Center and Center for Animal Resources and Development at Kumamoto University, Respectively. NA indicates not available.

| Gene symbol | Gene Name | RBRC No. | CARD ID |
|---|---|---|---|
| *AY761185* | cDNA sequence AY761185 | 12539 | NA |
| *Cimip2c* | ciliary microtubule inner protein 2C | 12226 | 3457 |
| *Cpsf4l* | cleavage and polyadenylation specific factor 4-like | 11979 | 3348 |
| *Defb25* | defensin beta 25 | 12538 | NA |
| *Gstt4* | glutathione S-transferase, theta 4 | 12345 | 3534 |
| *Pom121l12* | POM121 membrane glycoprotein-like 12 | 12537 | NA |
| *Pom121l2* | POM121 transmembrane nucleoporin like 2 | 12536 | NA |
| *Prss42, Prss43, Prss44 (Triple KO)* | serine protease 42<br>serine protease 43<br>serine protease 44 | 12306 | NA |
| *Tas2r119* | taste receptor, type 2, member 119 | 12572 | NA |
| *Tmem144* | transmembrane protein 144 | 12461 | 3560 |
| *Wdr27* | WD repeat domain 27 | 12304 | NA |
| *Wdr95* | WD40 repeat domain 95 | 12305 | NA |

**Supplementary Table 2.** primer information for RT-PCR

| Gene symbol | Primer sequences | Amplified DNA products (bp) |
|---|---|---|
| *Tas2r119* | Fw: gcaaatggcctcattgtggt<br>Rv: agggcatgtctgctaggttc | 596 |
| *Gapdh* | Fw: tgaagcaggcatctgaggg<br>Rv: cgaaggtggaagagtgggag | 102 |

**Supplemental Table 3.**

Two guide RNAs targeting the upstream and downstream regions of each gene were used to generate gene-deficient mice. The efficiency of embryo transplantation was represented by the number of total pups delivered by pseudo-pregnant mice divided by the number of total embryos used for oviduct transplantation. The efficiency of genome editing was determined by the number of pups carrying gene deletion divided by the number of pups subjected to genotyping. We performed Sanger sequencing analysis of the deletion allele and identified the deleted sequence information and DNA length in each gene-deficient line.

**Supplemental Table 4.**

The sequence information of primers and PCR conditions used for genotyping in each line.
